# Supplementary material for: Trimodal Single-Cell Gene Regulatory Networks Reveal Principles of Stemness Loss and Cell Fate Acquisition in Human Hematopoiesis
Source: bioRxiv. 2025 Sep 16:2025.09.11.675740. Preprint. [Version 1] doi: 10.1101/2025.09.11.675740 (PMC12458473; doi:10.1101/2025.09.11.675740)
Supplement: Supplement 2 [file NIHPP2025.09.11.675740v1-supplement-2.pdf]

bioRxiv preprint doi: https://doi.org/10.1101/2025.09.11.675740; this version posted September 16, 2025. The copyright holder for this preprint (which was not certified by peer review) is the author/funder, who has granted bioRxiv a license to display the preprint in perpetuity. It is made available under aCC-BY 4.0 International license.

SUPPLEMENTAL INFORMATION

Supplemental Data S1. Zipped file containing a "README.docx" file with installation instructions for the BioTapestry desktop application, and "AllBTPFiles.zip" file containing cell-specific GRNs in BioTapestry format.

Table S1. Antibodies used in TEAseq.

Table S2. Differentially expressed surface proteins across populations (lists extracted from MultiVI outputs).

Table S3. Differentially expressed genes across populations (lists extracted from MultiVI outputs).

Table S4. Full MultiVI differential expression statistics for genes and proteins.

Table S5. Enhancer-based gene regulatory networks (constructed with SCENIC+).

Table S6. TEAseq quality control metrics.

SUPPLEMENTARY FIGURES

bioRxiv preprint doi: https://doi.org/10.1101/2025.09.11.675740; this version posted September 16, 2025. The copyright holder for this preprint (which was not certified by peer review) is the author/funder, who has granted bioRxiv a license to display the preprint in perpetuity. It is made available under aCC-BY 4.0 International license.

FIGURE S1

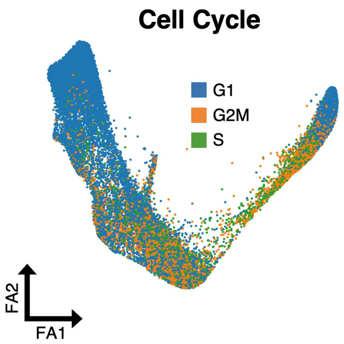

Figure S1 (related to Figure 1). Trimodal Profiling of Human Early Hematopoietic Differentiation.

FA map of the MultiVI latent space (23,887 cells) colored by cell cycle phase.

bioRxiv preprint doi: https://doi.org/10.1101/2025.09.11.675740; this version posted September 16, 2025. The copyright holder for this preprint (which was not certified by peer review) is the author/funder, who has granted bioRxiv a license to display the preprint in perpetuity. It is made available under aCC-BY 4.0 International license.

FIGURE S2

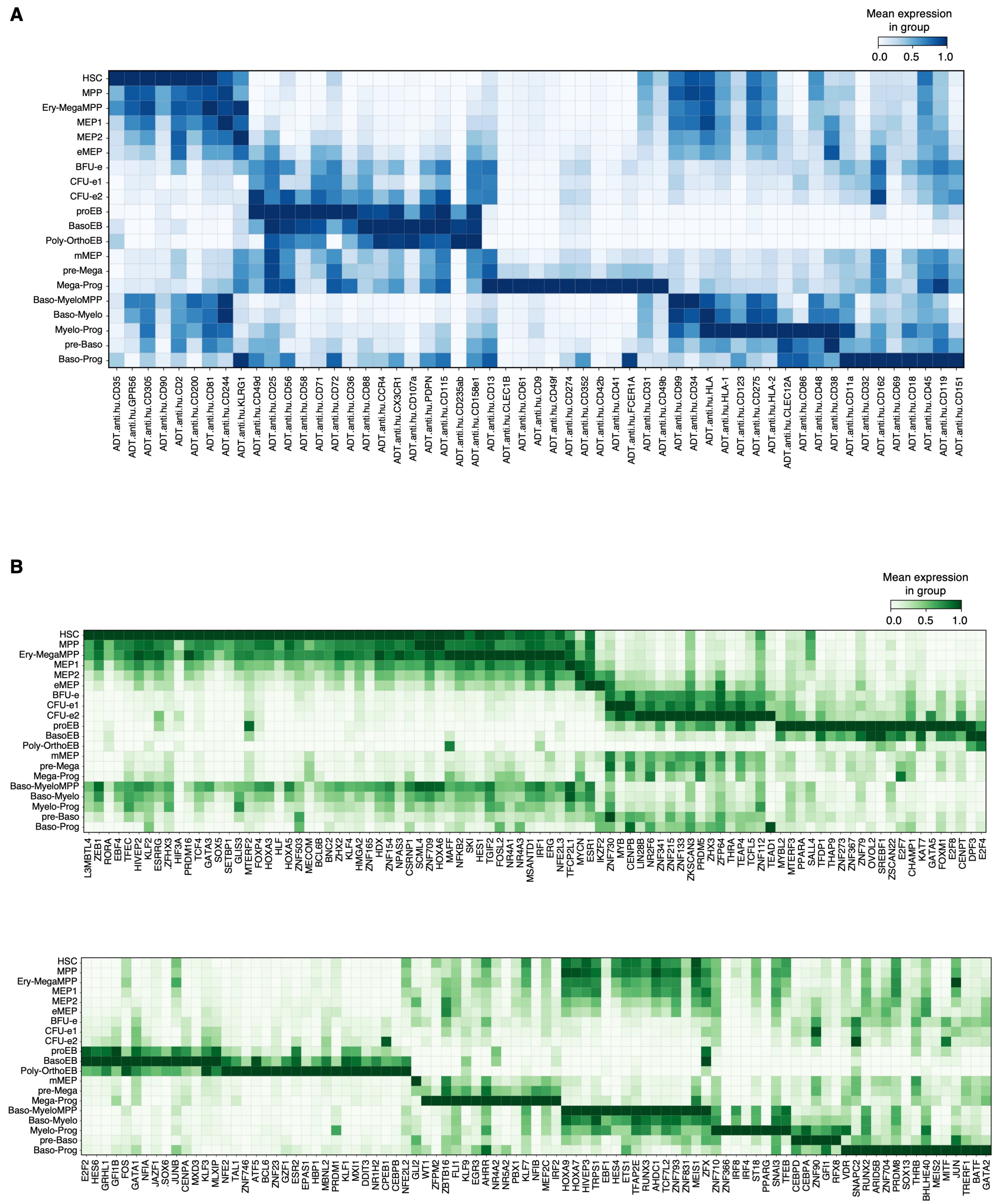

Figure S2 (related to Figure 2). Surface Proteins and RNA Markers of Hematopoietic Populations.

bioRxiv preprint doi: https://doi.org/10.1101/2025.09.11.675740; this version posted September 16, 2025. The copyright holder for this preprint (which was not certified by peer review) is the author/funder, who has granted bioRxiv a license to display the preprint in perpetuity. It is made available under aCC-BY 4.0 International license.

(A) Heatmap of selected surface protein markers identified from one-versus-all differential analyses. X-axis: surface proteins; Y-axis: populations defined in Fig. 2B. See Table S2 for the full list of markers.

(B) Heatmap of selected RNA markers identified from one-versus-all differential analyses. X-axis: genes; Y-axis: populations defined in Fig. 2B. See Table S3 for the full list of markers.

See also Table S4 for the complete results of one-versus-all tests.

bioRxiv preprint doi: https://doi.org/10.1101/2025.09.11.675740; this version posted September 16, 2025. The copyright holder for this preprint (which was not certified by peer review) is the author/funder, who has granted bioRxiv a license to display the preprint in perpetuity. It is made available under aCC-BY 4.0 International license.

FIGURE S3

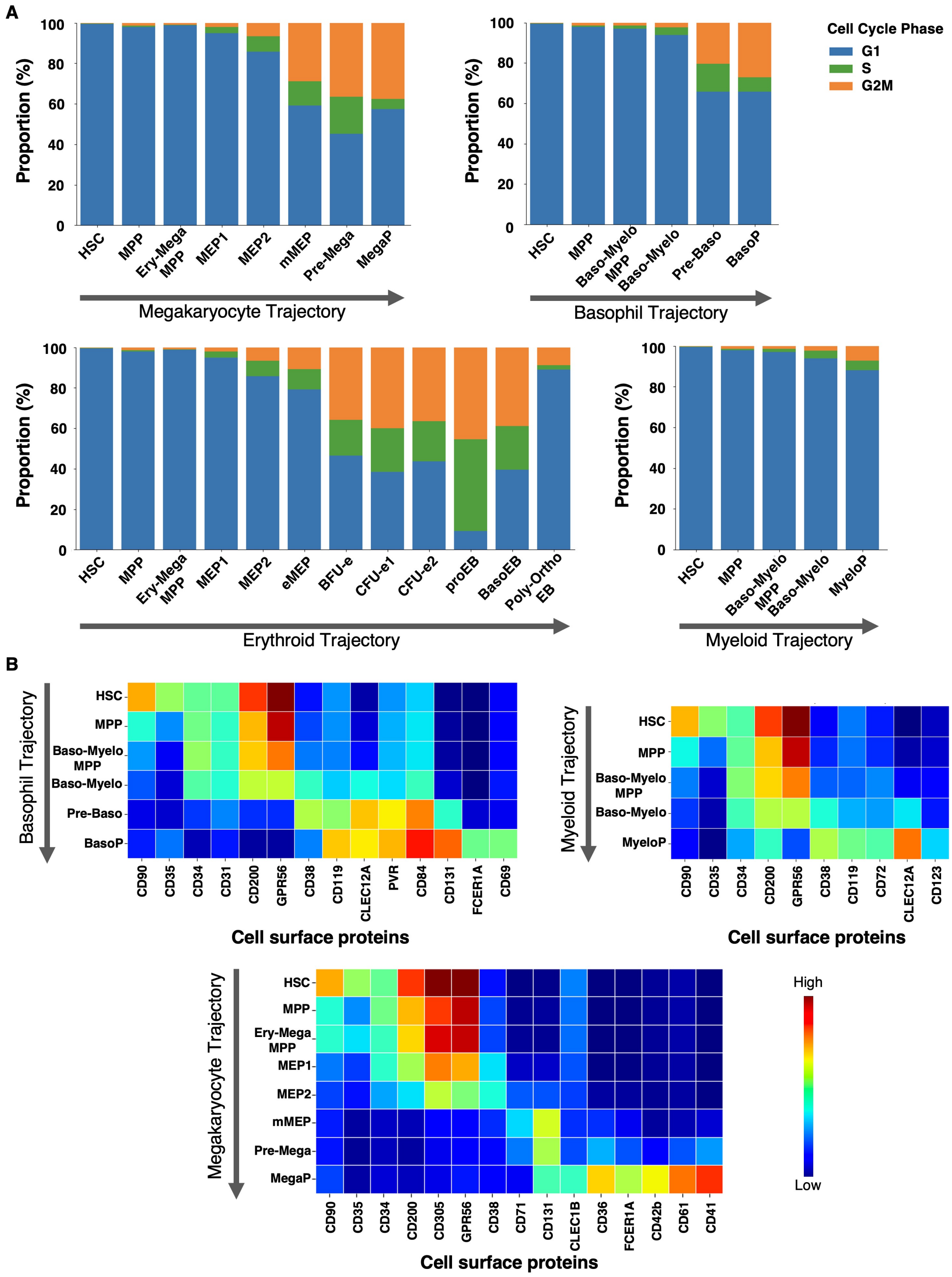

Figure S3 (related to Figure 2). Cell Cycle Distribution and Surface Protein Profiles of Hematopoietic Populations.

bioRxiv preprint doi: https://doi.org/10.1101/2025.09.11.675740; this version posted September 16, 2025. The copyright holder for this preprint (which was not certified by peer review) is the author/funder, who has granted bioRxiv a license to display the preprint in perpetuity. It is made available under aCC-BY 4.0 International license.

(A) Bar graphs showing the proportion of cells in each cell-cycle phase across populations along hematopoietic trajectories.

(B) Heatmap of selected surface protein expression across populations along the indicated trajectories. X-axis: surface proteins; Y-axis: populations defined in Fig. 2B. Color scale: TotalVI-normalized protein abundance, from low (blue) to high (red).

bioRxiv preprint doi: https://doi.org/10.1101/2025.09.11.675740; this version posted September 16, 2025. The copyright holder for this preprint (which was not certified by peer review) is the author/funder, who has granted bioRxiv a license to display the preprint in perpetuity. It is made available under aCC-BY 4.0 International license.

FIGURE S4

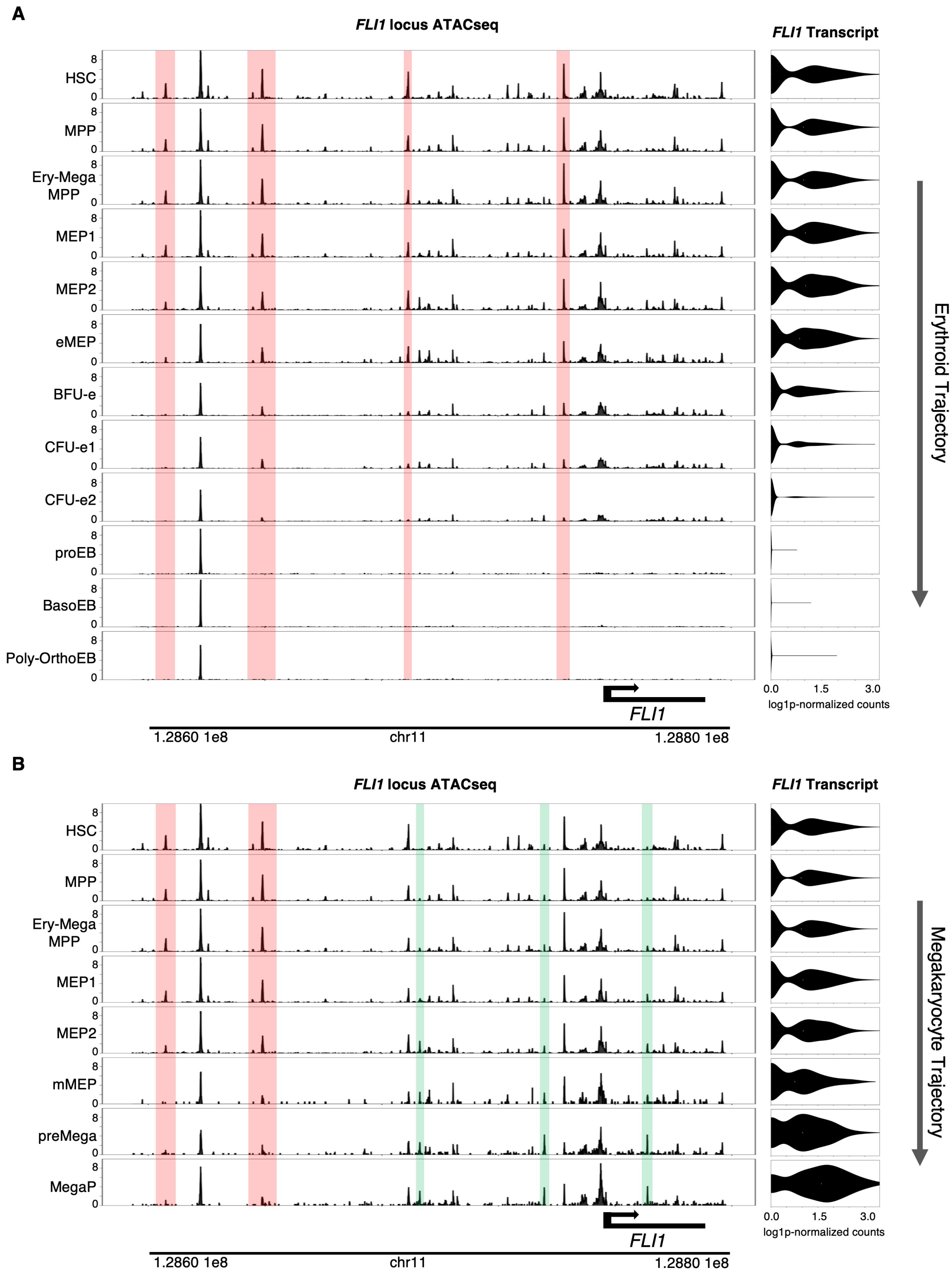

Figure S4 (related to Figure 5). Complex Changes in Enhancer Activity at the FLI1 Locus along Hematopoietic Trajectories

bioRxiv preprint doi: https://doi.org/10.1101/2025.09.11.675740; this version posted September 16, 2025. The copyright holder for this preprint (which was not certified by peer review) is the author/funder, who has granted bioRxiv a license to display the preprint in perpetuity. It is made available under aCC-BY 4.0 International license.

(A) Along the erythroid trajectory, progressive decrease in FLI1 expression is accompanied by gradual closure of several enhancers (highlighted in pink). Chromatin accessibility is shown as ATAC-seq tracks (left), and transcript levels as violin plots (right), in the indicated cell types (as defined in Fig. 2B).

(B) Along the megakaryocyte trajectory, progressive increase in FLI1 expression is accompanied by gradual opening of some enhancers (highlighted in green) and closure of others (highlighted in pink). Chromatin accessibility is shown as ATAC-seq tracks (left), and transcript levels as violin plots (right), in the indicated cell types (as defined in Fig. 2B).

Tracks were downloaded from our web application (https://hematopoiesis.systemsbiology.net).

bioRxiv preprint doi: https://doi.org/10.1101/2025.09.11.675740; this version posted September 16, 2025. The copyright holder for this preprint (which was not certified by peer review) is the author/funder, who has granted bioRxiv a license to display the preprint in perpetuity. It is made available under aCC-BY 4.0 International license.

FIGURE S5

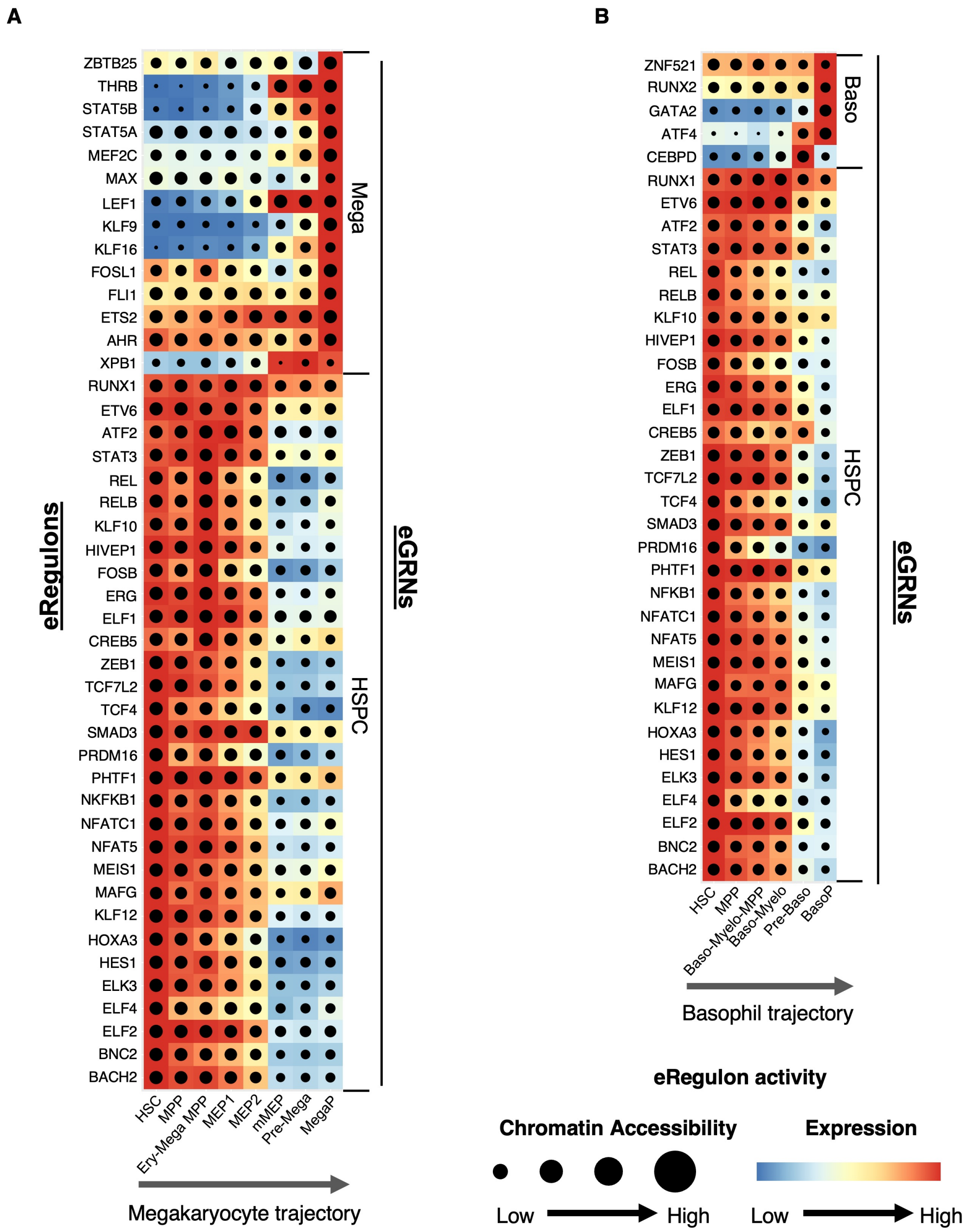

Figure S5 (related to Figure 6). Rewiring of Cell-Specific eGRNs along Hematopoietic Trajectories

Heatmap / dot-plot of eRegulon activity generated with SCENIC+. Each eRegulon consists of an effector TF (listed on the left), the CREs (enhancers and promoters) bound by that TF, and its predicted target genes. Target gene expression is

bioRxiv preprint doi: https://doi.org/10.1101/2025.09.11.675740; this version posted September 16, 2025. The copyright holder for this preprint (which was not certified by peer review) is the author/funder, who has granted bioRxiv a license to display the preprint in perpetuity. It is made available under aCC-BY 4.0 International license.

indicated by color intensity, and the fraction of CREs with open chromatin is represented by dot size. Populations (x-axis) are ordered along the megakaryocyte (A) and basophil (B) trajectories (as defined in Fig. 2B). Selected eRegulons are shown on the y-axis (left), grouped into eGRNs (right).

See Table S5 for the full list of TFs and target genes.

Data are available for interactive exploration in our web application (https://hematopoiesis.systemsbiology.net) and in BioTapestry format (Supplemental Data S1).

bioRxiv preprint doi: https://doi.org/10.1101/2025.09.11.675740; this version posted September 16, 2025. The copyright holder for this preprint (which was not certified by peer review) is the author/funder, who has granted bioRxiv a license to display the preprint in perpetuity. It is made available under aCC-BY 4.0 International license.

FIGURE S6

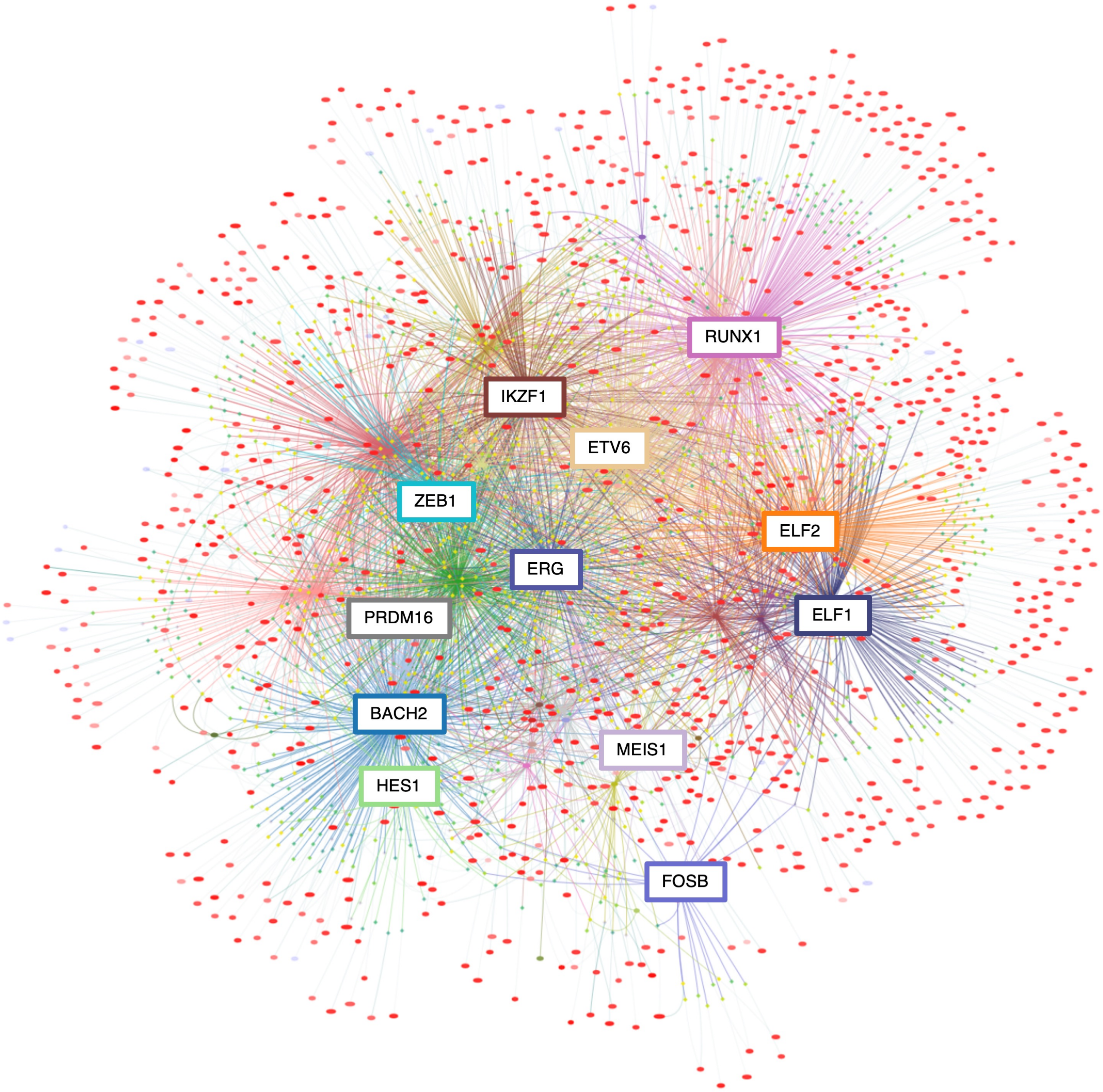

Figure S6 (related to Figure 6). HSPC Enhancer-Based Gene Regulatory Network.

Selected eRegulons from the HSPC eGRN are shown. Effector TFs are highlighted in colored boxes. Circles represent target genes (expression shown on a red [high] to blue [low] scale), and diamonds represent cis-regulatory elements (promoters

bioRxiv preprint doi: https://doi.org/10.1101/2025.09.11.675740; this version posted September 16, 2025. The copyright holder for this preprint (which was not certified by peer review) is the author/funder, who has granted bioRxiv a license to display the preprint in perpetuity. It is made available under aCC-BY 4.0 International license.

and enhancers; chromatin accessibility shown on a yellow [high] to dark blue [low] scale). For clarity, only the top 4,000 most variable target genes are displayed. See Table S5 for the full list TFs and target genes. The eGRN shown here was retrieved from our interactive web application (https://hematopoiesis.systemsbiology.net). All networks, including this one, are available both online for interactive exploration and as BioTapestry files in Supplemental Data S1.

bioRxiv preprint doi: https://doi.org/10.1101/2025.09.11.675740; this version posted September 16, 2025. The copyright holder for this preprint (which was not certified by peer review) is the author/funder, who has granted bioRxiv a license to display the preprint in perpetuity. It is made available under aCC-BY 4.0 International license.

FIGURE S7

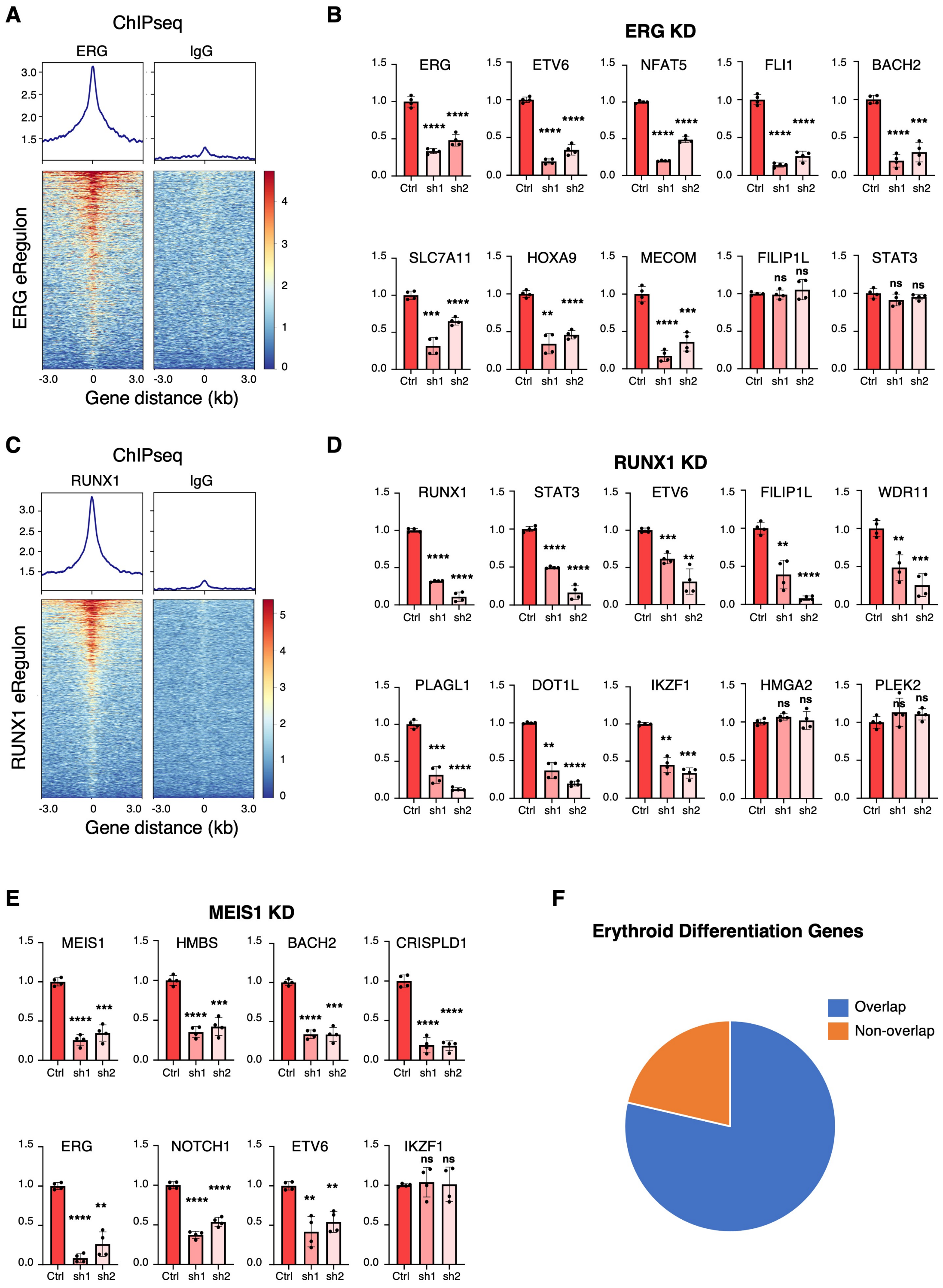

Figure S7 (related to Figure 6). Experimental Validation of Enhancer-Based Gene Regulatory Networks.

bioRxiv preprint doi: https://doi.org/10.1101/2025.09.11.675740; this version posted September 16, 2025. The copyright holder for this preprint (which was not certified by peer review) is the author/funder, who has granted bioRxiv a license to display the preprint in perpetuity. It is made available under aCC-BY 4.0 International license.

(A) ERG binds to predicted target CREs within the ERG eRegulon. Published ChIP-seq data [91] showing ERG occupancy at predicted sites are displayed as a heatmap; IgG ChIP-seq served as a control.

(B) ERG activates its predicted target genes as part of the ERG eRegulon. ERG knockdown (KD) was induced by lentiviral delivery of two shRNAs (sh1, sh2) in $CD34^+$ HSPCs. FILIP1L and STAT3 were used as negative controls.

(C) RUNX1 binds to predicted target CREs within the RUNX1 eRegulon. Published ChIP-seq data [91] showing RUNX1 occupancy at predicted sites are displayed as a heatmap; IgG ChIP-seq served as a control.

(D) RUNX1 activates its predicted target genes as part of the RUNX1 eRegulon. KD was induced as in (B). HMGA2 and PLEK2 were used as negative controls.

(E) MEIS1 activates its predicted target genes as part of the MEIS1 eRegulon. KD was induced as in (B). IKZF1 was used as a negative control.

(F) Overlap between genes essential for erythroid differentiation (identified in a published genome-wide CRISPR screen [95]) and erythroid-specific eGRNs.

(A, C) Signal intensity represents RPKM-normalized ChIP-seq coverage.

(B, D, E) Expression of predicted target genes shown by qRT-PCR relative to GAPDH (mean ± SD, n=4). Two-tailed t test: ns, not significant ($p > 0.05$); *$p \leq 0.05$; **$p \leq 0.01$; ***$p \leq 0.001$; ****$p \leq 0.0001$.
